# Computed tomography features and clinical characteristics of gastritis cystica profunda

**DOI:** 10.1186/s13244-021-01149-5

**Published:** 2022-01-24

**Authors:** Rui Wang, Hao Lu, Juan Yu, Wenpeng Huang, Jing Li, Ming Cheng, Pan Liang, Liming Li, Huiping Zhao, Jianbo Gao

**Affiliations:** 1 Department of Radiology, The First Affiliated Hospital of Zhengzhou University, No. 1, East Jianshe Road, Zhengzhou, 450052 Henan China; 2 Henan Key Laboratory of Image Diagnosis and Treatment for Digestive System Tumor, No. 1, East Jianshe Road, Zhengzhou, 450052 Henan China; 3 Department of Radiology, The Affiliated Cancer Hospital of Zhengzhou University (Henan Cancer Hospital), No. 127, Dongming Road, Zhengzhou, 450008 China; 4 Department of Medical Information, The First Affiliated Hospital of Zhengzhou University, No. 1, East Jianshe Road, Zhengzhou, 450052 Henan China

**Keywords:** Stomach, Gastritis cystica profunda, Tomography (X-ray computed), Diagnosis

## Abstract

**Background:**

The diagnostic evidence of gastritis cystica profunda (GCP) are not adequately described due to its extremely low morbidity. This study aimed to analyze and summarize the comprehensive CT features and clinical characteristics of patients with GCP.

**Results:**

Nineteen patients were enrolled, including eight men and eleven women, with a mean age of 55.53 years. Only one patient had the history of gastric polypectomy. Among the nineteen cases, two cases were in the gastric cardia, four in the gastric fundus, eight in the gastric body and five in the gastric antrum. The shapes were sphere in thirteen patients, hemisphere in five patients and diffuse in one patient. The mean size of eighteen local lesions was 1.63 cm. The cystic changes in submucosa were detected in fifteen patients. Compared with the pancreas, most GCP lesions were hypo-attenuated on unenhanced CT (*n* = 8), in arterial phase (AP) (*n* = 17) and venous phase (VP) (*n* = 11). Fifteen patients had the peak enhancement in VP and two in AP. The rim-like enhancement with central low attenuation was clearly observed in thirteen patients. For the GCP accompanied by adenocarcinoma, the enhancement peak was present in AP and the gradual expansion of enhancement area was in VP. All patients underwent surgical or endoscopic resection. Sixteen cases had remission of symptoms and no recurrence.

**Conclusions:**

The careful analysis of CT features and clinical characteristics can provide support for deepening the understanding of the GCP. However, a more accurate diagnosis depends on histopathological features.

## Key points

- The common features of the GCP can be observed on CT images.
- Cystic changes and rim-like enhancement are the valuable CT features.
- Specific enhancement pattern may indicate the malignancy nature of GCP.

## Background

Gastritis cystica profunda (GCP) is a relatively rare disease characterized by hyperplasia and cystic dilation of gastric glands involving the submucosal layer or even muscularis propria of the stomach [1]. The term “GCP” was proposed based on the process in the stomach resembled the similar manner occurring in the colon [2]. Although the exact pathogenesis mechanism of GCP remains not well established, GCP is also thought to be associated with ischemia and chronic inflammation as well as defects in the mucosa [3]. The mucosal defects may be caused by surgery, biopsy and polypectomy, which promotes mucosal prolapse and herniation of glands into the submucosa. For the patients with un-operated stomach, widespread chronic active/atrophic gastritis are considered as an important factor leading to GCP [4]. In clinical, GCP is more likely to be underdiagnosed as the result of unremarkable clinical characteristics and nonspecific imaging manifestations. In addition, GCP has been considered to be a possible premalignant lesion in several reports [5–8]. Therefore, accurate diagnosis of GCP is very useful for the development of individualized treatment strategies.

Up to now, some cases of GCP have been reported in the previous medical literatures. These studies mostly focused on the clinical and pathological characteristics [2, 9, 10], but the systematic imaging features have not been discussed. The characteristic imaging findings could be conducive to the comprehensive evaluation of lesions, so as to avoid the unnecessary treatment. Both endoscopic ultrasonography (EUS) and computed tomography (CT) examination are relatively valuable in evaluating the gastric lesion. By EUS, GCP demonstrated anechoic, mixed heterogeneous with thickened mucosa and hypoechoic with microcysts [11, 12]. Although EUS exhibits a great use in depicting shape, extent and echoic patterns of the GCP, the invasive operation and low patient compliance in this complex procedure deserve our attention. CT examination also has some advantages in the diagnosis of the GCP, as it can perform noninvasive assessment of primary lesions and adjacent structures [13]. In addition, due to the characteristics of time-saving operation, low price and multi-parametric imaging, CT has been widely used to evaluate gastric lesions. Therefore, CT is a major diagnostic modality for gastrointestinal diseases and an indispensable supplement of EUS.

The purpose of our study was to analyze detailed CT findings and clinical manifestations of nineteen patients with GCP. And the valuable CT features and clinical characteristics were summarized to help prevent a potential misdiagnosis before treatment. Careful attention to comprehensive imaging observations and clinical diagnosis may have significant implications on reasonable therapy.

## Methods

### Patients

This study was approved by our institutional review board and the requirement for written informed consent was waived. From January 2013 to September 2021, a total of twenty-five GCP patients confirmed by pathology were searched initially from our institution. After excluding the patients with incomplete imaging data (*n* = 3), insufficient stomach distension (*n* = 2), and invisible lesion on CT images (*n* = 1), nineteen cases with GCP were finally reviewed. All enrolled patients had been confirmed as GCP by surgical or gastroscopic pathological examination and had undergone abdominal enhanced CT. The complete clinicopathologic data and pretreatment CT images were obtained from electronics databases.

### Clinical data and pathological evaluation

All patients underwent surgical or endoscopic excision of their gastric lesions. The clinical data were recorded for patients’ sex, age, symptom and duration, treatment history, hypertension, smoking, fecal occult blood test, tumor markers and operation. The tumor markers including carcinoembryonic antigen (CEA), alpha fetoprotein (AFP), carbohydrate antigen 125 (CA125) and carbohydrate antigen 199 (CA199). Additionally, the hematoxylin and eosin-stained slid were reviewed by an experienced gastrointestinal pathologist, who was blinded to the clinical or endoscopic information. Immunohistochemistry analysis of paraffin-embedded sections was performed if necessary. The follow-up period and recurrence were obtained from physicians or telephone interviews. The last follow-up time was September 2021.

### CT image acquisition

All patients underwent unenhanced and dual-phase contrast-enhanced CT examination. CT images were acquired with a 64-channel multi-detector CT scanner (Discovery CT750 HD CT Scanner, GE Healthcare Milwaukee, WI, USA) or a 16-channel multi-detector CT scanner (Brilliance 16, Philips Medical Systems, Cleveland, OH, USA). The main imaging parameters were as follows: detector collimation, 0.625 mm or 1.5 mm; pitch, 1.375:1 or 1.25:1; tube voltage, 120 kVp; tube current, 80–270 mAs; rotation time, 0.5–0.6 s; reconstruction section thickness, 5 mm and 1.25 mm. After fasting overnight, preparations were required for patient before CT scanning, including an injection of 20 mg of butyl scopolamine for decreasing gastrointestinal peristalsis and facilitating hypotonia and oral administration of 600–1000 mL of water to distend the stomach. For enhanced CT scans, 70–120 mL of iodinated contrast agent (350 or 370 mg I/mL) was injected at a flow rate of 3.0–3.5 mL/s via a peripheral vein with a dual high-pressure syringe. The enhanced CT images in arterial phase (AP) and venous phase (VP) were obtained with a scanning delay of 30 s and 70 s after the intravenous injection of contrast agent. The coronal and sagittal CT images were reconstructed by the multiplanar reformation technique.

### Image analysis

All images were transferred to ADW4.7 workstation (GE Healthcare, Milwaukee, WI, USA) for image analyzing. Two experienced radiologists (with 5 and 10 years of experience in abdominal radiology) reviewed the CT images of GCP by consensus with a standard abdominal window (width 220 HU, level 40 HU). They were blinded to the clinical information except for awareness of the gastric lesion. The radiological features of the GCP were as follows: location, shape (sphere, hemisphere or diffuse), pedicle, size of lesion (long-axis diameter), cystic change, attenuation of cystic area, separation in cysts, attenuation of solid lesions (hypo-, iso- or hyper-), enhancement degree (mild, moderate or obvious), peak enhancement phase (AP, VP or both), rim-like enhancement and imaging diagnosis. Cystic change was determined as liquid density area without enhancement on CT images. The attenuation of solid lesion was interpreted by comparing the density of gastric lesions and pancreatic parenchyma on non-enhanced and enhanced CT images. Enhancement degree was determined by the difference in attenuation of dynamic CT imaging, which classified as mild (< 20 HU), moderate (20-40 HU) and obvious enhancement (> 40 HU). The rim-like enhancement was evaluated based on enhanced images. When evaluating the solid area of GCP, we placed the region of interest at the largest cross-sectional area of the lesion and avoided the areas of cystic change and vessels. The measurements of quantitative features were repeated three times at same section and the average values of them were calculated to ensure reliability. Furthermore, CT findings were analyzed and compared with the clinical and pathological data.

## Results

### Clinical characteristics and follow-up data

Clinical characteristics and follow-up data of nineteen patients were presented in Table 1. All enrolled patients consisted of eight men (mean age 53.75 years; age range 30–67 years) and eleven women (mean age 56.82 years; age range 32–76 years). The mean age of the entire population was 55.53 years. Among nineteen patients, most patients were admitted with nonspecific symptoms, including epigastric pain or distension, dysphagia, nausea or vomiting and abnormal defecation. Four patients (No. 7, No. 11, No. 15 and No. 16) showed noticeable symptoms including acid reflux, hematemesis and melena. While the other two patients (No. 6 and No. 18) did not have any gastrointestinal symptoms and their lesions of stomach were occasionally detected. The symptom duration of these patients (before hospital visit) ranged from 1 to 365 days, with a median of 15 days. None of patients had the history of gastrectomy or gastroenterostomy, and only one of them (No. 4) underwent gastric polypectomy 20 years ago. Three patients had the history of hypertension and one patient had notable social history of smoking. Fecal occult blood test revealed positive expression in five patients and negative expression in nine patients. Laboratory findings showed that tumor markers (CEA, AFP, CA125 and CA199) were unremarkable. Operations were performed in all nineteen patients, including total gastrectomy (*n* = 1), subtotal gastrectomy (*n* = 3), endoscopic mucosal resection (EMR) (*n* = 7) and endoscopic submucosal dissection (ESD) (*n* = 8). There were no complications during their hospital stay, and they were discharged from the hospital 2–18 days post-operation. The median follow-up period were 31 months, ranging from 6 to 93 months. Sixteen cases showed that their symptoms relieved and no recurrence until the end of the follow-up period.

**Table 1:** Clinical characteristics and follow-up data of 19 patients with GCP

No	Sex	Age	Symptom and duration	Treatment history	Hyper-tension	Smoking	Fecal occult blood test	CEA	AFP	CA125	CA199	Operation	Pathologic diagnosis	Follow-up period (months)	Recurrence
1	F	32	Epigastric pain or distension 10D	−	−	−	−	−	−	−	−	Gastrectomy	GCP	93	−
2	M	64	Epigastric pain or distension 60D	−	−	−	+	−	−	−	−	ESD	GCP with low-grade epithelial dysplasia	69	−
3	F	55	Epigastric pain or distension 20D	−	−	−	NA	−	−	−	−	Gastrectomy	GCP	63	−
4	F	61	Dysphagia 15D	Gastric polypectomy	−	−	−	−	−	−	−	Gastrectomy	GCP	46	−
5	F	44	Nausea or vomiting 92D	−	−	−	NA	−	−	NA	−	ESD	GCP with heterotopic pancreas	46	−
6	F	62	Detect lesions by accident 1D	−	−	−	−	−	−	−	−	EMR	GCP	38	−
7	M	67	Dysphagia and acid reflux 30D	−	−	+	−	−	−	−	−	ESD	GCP	42	−
8	F	64	Nausea or vomiting 5D	−	−	−	+	−	−	−	−	EMR	GCP with high-grade epithelial dysplasia	39	NA
9	F	57	Nausea or vomiting 120D	−	−	−	−	−	−	−	−	ESD	GCP	37	NA
10	F	49	Epigastric pain or distension 20D	−	−	−	−	−	−	−	−	EMR	GCP	31	−
11	M	54	Hematemesis and melena 15D	−	−	−	+	−	NA	−	−	Gastrectomy	GCP	30	−
12	M	53	Abnormal defecation 6D	−	−	−	−	−	NA	−	−	EMR	GCP	20	−
13	F	70	Abnormal defecation 30D	−	+	−	+	−	−	−	−	ESD	GCP	17	−
14	F	76	Epigastric pain or distension 60D	−	+	−	NA	−	−	−	−	EMR	GCP	15	−
15	F	55	Hematemesis 10D	−	+	−	NA	−	−	−	−	EMR	GCP	13	NA
16	M	30	Acid reflux and heartburn 30D	−	−	−	+	−	−	−	NA	ESD	GCP	12	−
17	M	34	Epigastric pain or distension 15D	−	−	−	NA	−	−	−	−	ESD	GCP accompanied by adenocarcinoma	8	−
18	M	67	Detect lesions by accident 9D	−	−	−	−	−	−	−	−	EMR	GCP	8	−
19	M	61	Epigastric pain or distension 5D	−	−	−	−	−	−	−	−	ESD	GCP	6	−

GCP, gastritis cystica profunda; M, male; F, female; CEA, carcinoembryonic antigen (positive defined as > 5 U/mL); AFP, alpha fetoprotein (positive defined as > 10 U/mL); CA125, carbohydrate antigen 125 (positive defined as > 35 U/mL); CA199, carbohydrate antigen 199 (positive defined as > 35 U/mL); ESD, endoscopic submucosal dissection; EMR, endoscopic mucosal resection; + , yes/present/positive; −, no/absent/negative; *NA,* not available

### Pathological features

All the GCP patients were confirmed on the basis of histopathologic diagnosis. The cut surface demonstrated the thickening of the mucosa and submucosa with many small cystic spaces containing viscous liquid. The finding of hemorrhage was observed only in patient No. 11. Calcification was not detected in all specimens. Microscopically, the common characteristics of these lesions were shown as cystic dilation of the foveolar glands, which penetrated the mucosa layer and infiltrated widely into the underlying submucosa, even into the muscularis propria. The surrounding interstitial fibrous of lesions proliferated with infiltration of many inflammatory cells. Notably, low-grade and high-grade epithelial dysplasia were identified in patient No. 2 and No. 8, respectively. GCP accompanied by adenocarcinoma was found in patient No. 17. In addition, pancreatic acinar cells were present in the submucosa of patient No. 5, which was diagnosed as GCP with heterotopic pancreas.

### Imaging findings

**Fig. 1:**
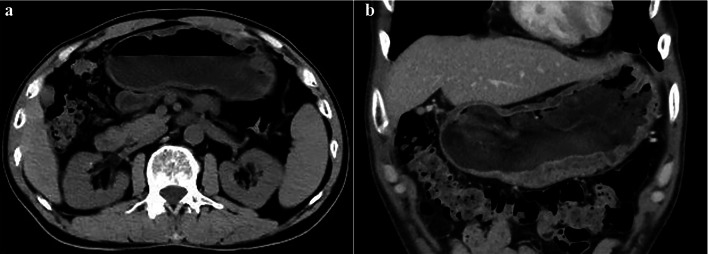
54-year-old man with GCP showing the diffuse thickening of gastric wall. **a** Axial unenhanced image reveals the thickened gastric mucosal folds pervading the large areas of gastric body. **b** Coronal venous phase CT shows multiple cysts in the submucosal layer

**Fig. 2:**
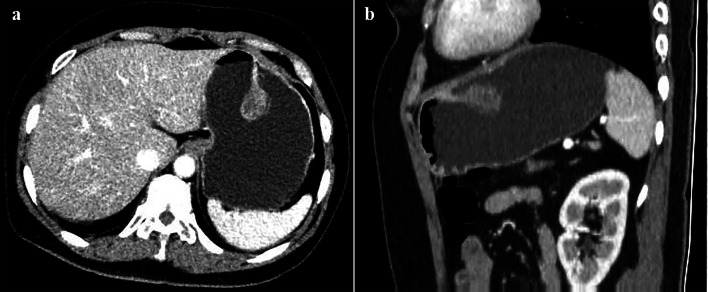
55-year-old woman with GCP revealing a 2.7-cm spherical mass with pedicle. Axial venous phase (**a**) and sagittal venous phase (**b**) CT images show a heterogeneous enhanced mass in the greater curvature of gastric body, which is connected to a 2.35-cm pedicle

The CT imaging features of nineteen cases with GCP were summarized in Table 2. Based on multiplanar reformation imaging, two cases (10.53%) were located in the gastric cardia, four (21.05%) in the gastric fundus, eight (42.10%) in the gastric body and five (26.32%) in the gastric antrum. The shapes of the GCP were sphere in thirteen patients (68.42%), hemisphere in five patients (26.32%) and diffuse in one patient (5.26%). The diffuse lesion showed as thickened gastric mucosal folds pervading the large areas of gastric body (Fig. 1). In addition, similar to some polyps, pedicle was found in five GCP cases with spherical lesions. The longest diameter of pedicle was 2.35 cm in patient No. 15 (Fig. 2). Except for diffuse lesion, the mean size of lesions in eighteen patients was 1.63 cm, ranging from 0.7 to 3.3 cm. Among these lesions, three cases (16.67%) of GCP were less than 1.0 cm, ten (55.56%) were 1.0 to 2.0 cm, and the remaining five (27.77%) were greater than 2.0 cm.

**Table 2:** Preoperative CT features for 19 patients diagnosed with GCP

No	Location	Shape	Pedicle	Size (cm)	Cystic change	Separation in cysts	Attenuation of cystic area (HU)	Attenuation of solid lesions			Enhancement degree		Peak enhancement phase	Rim-like enhancement	Imaging diagnosis
								Unenhanced	AP	VP	AP	VP			
1	Fundus	Sphere	−	2.4	+	−	23	Hypo	Hypo	Hypo	Mild	Moderate	VP	+	GST
2	Antrum	Sphere	−	1.3	+	−	32	Hyper	Hypo	Hypo	Mild	Moderate	VP	−	Adenoma
3	Body	Sphere	−	1.2	+	−	28	Hypo	Hypo	Iso	Mild	Obvious	VP	+	GST
4	Antrum	Sphere	−	3.3	+	+	24	Iso	Hypo	Iso	Moderate	Obvious	VP	+	GST
5	Antrum	Sphere	−	2.3	+	−	16	Iso	Hypo	Hypo	Mild	Moderate	VP	+	GST
6	Body	Sphere	−	1.2	+	−	23	Hypo	Hypo	Hypo	Mild	Moderate	VP	+	GST
7	Cardia	Hemisphere	−	2.6	+	+	22	Iso	Hypo	Hypo	Mild	Moderate	VP	+	Submucosal mass
8	Fundus	Sphere	+	0.8	−	−	−	Hypo	Iso	Hypo	Obvious	Moderate	AP	−	Polyp
9	Cardia	Hemisphere	−	1.9	+	−	28	Hypo	Hypo	Hyper	Moderate	Obvious	VP	+	GST
10	Body	Sphere	−	1.2	+	−	25	Hyper	Iso	Hyper	Mild	Obvious	VP	+	Submucosal mass
11	Body	Diffuse	−	−	+	−	45	Hyper	Hypo	Hypo	Moderate	Obvious	VP	−	GL
12	Antrum	Sphere	+	1.2	+	−	17	Hyper	Hypo	Hyper	Mild	Moderate	VP	−	Polyp
13	Body	Sphere	−	0.9	−	−	−	Hypo	Hypo	Hypo	Moderate	Obvious	VP	−	GST
14	Fundus	Sphere	+	1.1	+	−	12	Iso	Hypo	Hypo	Mild	Moderate	VP	+	Polyp
15	Body	Sphere	+	2.7	+	+	24	Iso	Hypo	Hypo	Moderate	Obvious	VP	+	Polyp
16	Antrum	Hemisphere	−	1.4	+	−	36	Iso	Hypo	Iso	Moderate	Moderate	Both	+	GST
17	Body	Hemisphere	−	1.1	−	−	−	Iso	Hypo	Iso	Obvious	Mild	AP	+	Submucosal mass
18	Body	Sphere	+	0.7	−	−	−	Hypo	Hypo	Iso	Obvious	Obvious	Both	−	GST
19	Fundus	Hemisphere	−	2.0	+	−	2	Hypo	Hypo	Hypo	Moderate	Obvious	VP	+	Submucosal mass

GCP, gastritis cystica profunda; AP, arterial phase; VP, venous phase; GST, gastric stromal tumor; GL, gastric lymphoma; +, yes/present/positive; −, no/absent/negative; NA, not available

**Fig. 3:**
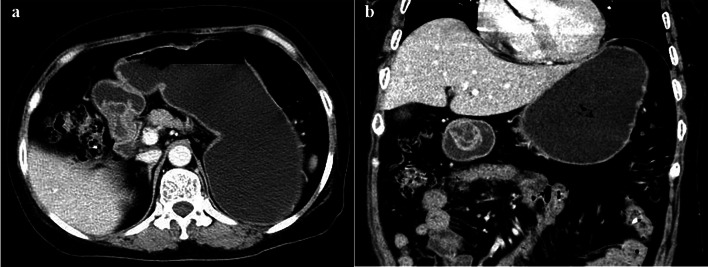
61-year-old woman with GCP showing a 3.3-cm cystic mass. Axial venous phase (**a**) and coronal venous phase (**b**) CT images show the cystic mass located in the gastric antrum. This lesion is featured as multiple cysts separated by fibrous

The cystic changes were detected on CT images in fifteen patients (78.95%). Most of cystic components presented heterogeneous density with the attenuation values ranging from 2 to 45 HU in the unenhanced phase. Among of them, multiple cysts separated by fibrous septum were visible in three patients. Additionally, multiple scattered cysts were distributed in the submucosal layer in patient No. 11 (Fig. 1). In relation to lesion size and cystic change, we found that the lesions with cystic change were almost larger than 1 cm. Specifically, the lesions with multiple cysts were lager than 2.5 cm (Fig. 3), while with single capsular space were not greater than 2.4 cm.

**Fig. 4:**
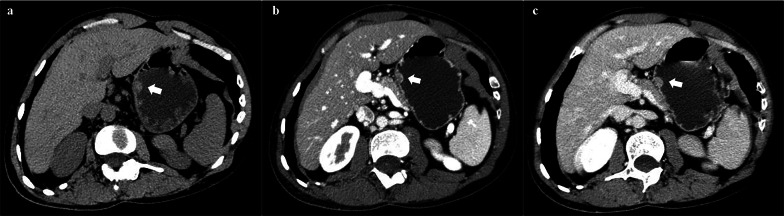
34-year-old man with GCP accompanied by adenocarcinoma. **a** Axial unenhanced CT reveals an iso-attenuating hemisphere mass (*arrow*) in the lesser curvature of gastric body. **b** Axial arterial phase CT shows the obvious rim-like enhancement with central low attenuation of this mass (*arrow*). **c** Axial venous phase CT shows mild enhanced mass (*arrow*). The enhancement area of this mass is gradually expanding compared to the rim-like enhancement in arterial phase

The attenuation of lesions was compared with that of the pancreatic parenchyma. The solid components showed hypoattenuation in unenhanced CT images in eight of nineteen patients (42.11%). And hypoattenuation was the most common both in AP (89.47%, 17/19) and VP (57.89%, 11/19). In terms of enhancement degree of lesions, nine lesions (47.37%) were mildly enhanced in AP. In VP, moderate enhancement was seen in nine lesions (47.37%) and obvious enhancement in other nine lesions (47.37%). Fifteen cases (78.94%) had the peak enhancement in VP and two cases (10.53%) in AP. The other two cases (10.53%) showed the similar enhancement degree in both phases. According to enhancement pattern of gastric wall, these lesions were mostly featured as intact mucosa with obvious enhancement, cystic capsule without enhancement in center and muscular layer with mild to moderate enhancement. Moreover, rim-like enhancement with central low attenuation was clearly observed in thirteen patients (68.42%). Interestingly, only in patient No. 17 with GCP accompanied by adenocarcinoma, rim-like enhancement was present only in AP and the enhancement area is gradually expanding in VP (Fig. 4). In the assessment of surrounding conditions, no evidence of lymph node involvement was found in any lesions.

Unfortunately, most of the lesions were misdiagnosed as other benign or malignant tumors before treatment. In our study, nine cases were misdiagnosed as gastric stromal tumor (GST), four cases as polyp, one case as gastric lymphoma (GL) and one case as adenoma. The remaining four cases were not definitive, and the radiologists preliminarily speculated that they were submucosal masses.

## Discussion

In our study, we systematically analyzed and summarized the CT findings and clinical manifestations of nineteen patients with GCP. Some new and meaningful findings are expected to supplement the diagnosis of GCP. This disease occurs more commonly in the elderly and could also occurs in a few young patients [4]. In our study, the age of GCP patients ranged from 30 to 76 years and the ratio of female to male is 11:8. Xu et al. [4] found a similar age range of GCP patients and a higher proportion of female. Some GCP patients had nonspecific gastrointestinal symptom or had no symptom, while others had unusual symptoms that deserve the attention of clinicians, including acid reflux, hematemesis and melena. Gastric obstruction could also be found in several reports [14, 15], but not in this study. It was often speculated that the GCP developed in patients who had undergone gastric surgery [16, 17]. However, more and more cases were reported in an unoperated stomach [18–20]. In our study, most cases (18/19, 94.74%) had no history of gastric surgery, suggesting that the mucosal defects caused by surgery may not be the major factor leading to GCP. Moreover, other medical history and laboratory examination had no considerable value for the diagnosis of GCP.

To our knowledge, GCP is generally benign and usually characterized by polypoid cystic ectasia of gastric glands involving the submucosa. Notably, a previous study suggested that downward adeno-cystic proliferation of glands was a dysplastic or precancerous change [21]. Furthermore, there have also been some reports of GCP associated with atypical hyperplasia and carcinoma development [6, 8, 22]. In the present cases, two cases were confirmed pathologically as epithelial dysplasia in the deeper part of lesions, which was reasonable to conclude that GCP might be a precursor of gastric cancer. Besides, patient No. 17 was identified as gastric adenocarcinoma arising in GCP, though he had no history of endoscopic procedures or surgery to cause mucosal injury. Microscopically, a small amount of glandular epithelium showed infiltrative growth, with positive expression of MUC5AC and MUC1. Iwanaga et al. [23] indicated that GCP accompanied 3.0% of gastric carcinoma cases in 1975, while more and more cases associated with malignancy have been reported recently. In addition, GCP with heterotopic pancreas was confirmed in patient No. 5, which is a rare extremely gastric submucosal lesion [24]. Consequently, it is necessary to perform the histopathologic confirmation when GCP is suspected.

To date, the standardized management strategy of GCP has yet no consensus. For the sake of maximizing patients’ benefit, multiple resection methods including EMR, ESD and gastrectomy were performed in our cases. The endoscopic resection or gastrectomy could be recommended depending on the lesion size and localization [19, 25, 26]. Fifteen patients (78.95%) underwent EMR or ESD in this study. In fact, endoscopic resection could not only effectively remove lesions but also preserve gastric function with minimal injury. Xu et al. [4] suggested that the combination of EUS and ESD made an open surgical procedure unnecessary. In our opinion, gastrectomy may be also essential in some cases. Considering the hemorrhage in gastric body and CT features of diffuse thickening of gastric mucosa, one patient (No. 11) underwent the total gastrectomy. Moreover, partial gastrectomy was performed in three patients in present study. In the report of one case with 2-year follow-up, GCP was recurred in just 6 months after surgical resection [26]. Since the history of gastric surgery is a significant etiologic factor, long surveillance of the risk of recurrence is essential. In our study, no recurrence was observed in any of the sixteen interviewees, and in particular, four surgical patients had a remarkable recovery.

From the previous and present imaging findings, the GCP could be found in every part of stomach and its common site was gastric body. The definite shapes of GCP could also be observed noninvasively through CT scans. Most lesions were sphere or hemisphere and some sphere lesions (5/13) had the pedicle with different lengths. Moreover, only one patient presented with swelling of gastric mucosal folds involving the whole gastric body, which can easily be misdiagnosed as gastric malignant tumor. In some case reports, the size of GCP lesions accompanied by malignancy varied from 0.4 to 6.0 cm [5–8, 22, 27]. Tatsuya et al. [28] have concluded that the GCP lesion greater than 5 cm suggested the risk of malignancy. In our study, the sizes of three cases with epithelial dysplasia or adenocarcinoma were not greater than 1.5 cm. Thus, the correlation between lesion size and malignancy was still controversial.

Cystic change was a significant and characteristic manifestation of GCP, which could be easily identified and evaluated on CT images in terms of attenuation, shape and size of cyst. In this study, cystic components were present in most of lesions (78.95%). The cystic change was not detected in four lesions, which may be related to the fact that these lesions were relatively small (0.7–1.1 cm) and the subtle cysts were difficult to be detected on CT images. By measuring the attenuation of cystic capsular space, we found their inconsistent attenuation, which are closely related to the composition and proportion of cystic fluid. A higher CT value indicated a higher content of mucus protein in the cystic fluid. Additionally, inflammatory cell deposition or intracapsular hemorrhage may be the reason for the hyper-attenuation of cystic fluid [29]. Multiple cysts and the separation of cystic lesion have been mentioned in previous researches of GCP [25, 30–32]. Similarly, this feature was observed in a few larger masses in our series. Based on this finding, GCP lesions larger than 2.5 cm were more likely to suggest multiple cysts separated by septum. Meanwhile, multiple cysts could also be found in diffuse lesion with thickened gastric mucosal folds. Similar results were obtained by Okada et al. [11], who reported the thickened stomach walls of GCP patients consist mostly of multiple cysts in the submucosal layer.

With regard to the attenuation of solid components, the low attenuation was most common in unenhanced CT images as well as in enhanced phase. Most lesions reached their enhancement peak in VP, suggesting the progression enhancement was a common enhancement pattern of GCP. Two cases (No. 8 and No. 17) had the enhancement peak in AP, and they were identified as GCP with high-grade epithelial dysplasia and GCP accompanied by adenocarcinoma, respectively. The attenuation in AP mainly reflects the capillary density and the blood supply of lesion [33]. When the enhancement degree of GCP is higher in AP, the possibility of dysplasia or carcinoma development should be noticed. Furthermore, peripheral rim-like enhancement was typically found in many cases. It might be composed of the markedly enhancing gastric mucosa, and the internal hypo-attenuated portion with cystic capsular space [34]. The only exception is the lesion of patient No. 17 with GCP accompanied by adenocarcinoma, who showed the rim-like enhancement only in AP and the expansion of enhancement area in VP. The gradually expanding enhancement area may reflected the balance of blood supply inside the cystic capsule.

To avoid aggressive treatment, it is necessary to make differential diagnosis between the GCP and the tumor with malignant potential, such as GST and GL. GSTs are a heterogeneous group of neoplasms exhibiting varying malignancy potential. They usually occur with similar frequency in males and females. Some small GSTs always have low or no mitotic activity, and thus the patients with these tumors usually have atypical or no clinical symptom, which is difficult to distinguish from GCP [35]. In the analysis of CT findings, GSTs presented as spherical or lobulated solid mass, moderate or obvious enhancement due to abundant blood supply, and intraluminal or extraluminal growth. When GCP lesions are characterized by thickened gastric mucosal folds pervading multiple regions of the gastric wall, primary GL could be considered in differential diagnosis of neoplasms in the stomach. The most common CT manifestation of GL is the diffuse thickening of the gastric wall or homogeneous soft tissue mass, with slight or similar attenuation compared to normal gastric wall. Furthermore, the widespread lymphadenopathy in the retroperitoneum or elsewhere in the abdomen should conduce to the diagnosis of lymphoma [36]. Other differential diagnoses were benign diseases, such as gastric polyp, gastric diverticulum, gastric duplication cyst and Ménétrier disease. Although the shape of GCP is similar to that of gastric polyp, cystic change can be regarded as the identification between them. In addition, the rim-like enhancement is of great significance in the diagnosis of GCP. Gastric diverticulum preferentially situates in gastric antrum. The gases and liquids are observed in the gastric cavity, indicating that the opening of the diverticulum is connected with the gastric cavity. Gastric duplication cyst is an extremely uncommon lesion, which is common in infants and sometimes accompanied by other malformations [37]. Ménétrier disease usually presents with thickened gastric folds, but without cystic changes.

## Conclusions

In this study, we analyzed the CT features and clinical characteristics of GCP to deepen the understanding of this rare gastric lesion. The clinical features of GCP are lack of specificity, and most patients had no history of gastric surgery. The cystic change in submucosa, progression enhancement and peripheral rim-like enhancement might be the valuable CT characteristics to distinguish the GCP from other lesions. Moreover, the enhancement peak in AP and gradual expansion of enhancement area in VP could help to identify the malignant nature of GCP. These characteristics can provide support for further research of GCP. However, what we concluded in this study should be verified in larger scale researches, and a more accurate diagnosis depends on histopathological features.

## Data Availability

The dataset used or analyzed during the current study are available from the corresponding author on reasonable request.

## Abbreviations

AFP: Alpha fetoprotein
AP: Arterial phase
CA125: Carbohydrate antigen 125
CA199: Carbohydrate antigen 199
CEA: Carcinoembryonic antigen
CT: Computed tomography
EMR: Endoscopic mucosal resection
ESD: Endoscopic submucosal dissection
EUS: Endoscopic ultrasonography
GCP: Gastritis cystica profunda
GL: Gastric lymphoma
GST: Gastric stromal tumor
VP: Venous phase

## References

[1] Littler ER, Gleibermann E (1972). Gastritis cystica polyposa. (Gastric mucosal prolapse at gastroenterostomy site, with cystic and infiltrative epithelial hyperplasia). Cancer.

[2] Franzin G, Novelli P (1981). Gastritis cystica profunda. Histopathology.

[3] Brandt LJ (2013). Gastritis cystica profunda with a long stalk. Gastrointest Endosc.

[4] Xu G, Peng C, Li X (2015). Endoscopic resection of gastritis cystica profunda: preliminary experience with 34 patients from a single center in China. Gastrointest Endosc.

[5] Park CH, Park JM, Jung CK (2009). Early gastric cancer associated with gastritis cystica polyposa in the unoperated stomach treated by endoscopic submucosal dissection. Gastrointest Endosc.

[6] Ogasawara N, Noda H, Kondo Y (2014). A case of early gastric cancer arising from gastritis cystica profunda treated by endoscopic submucosal dissection. Case Rep Gastroenterol.

[7] Huang D, Zhan Q, Yang S, Sun Q, Zhou Z (2018). Synchronous double superficial mixed gastrointestinal mucus phenotype gastric cancer with gastritis cystica profunda and submucosal lipoma: a case report. Medicine (Baltimore).

[8] Wahi J, Pagacz M, Ben-David K (2020). Gastric adenocarcinoma arising in a background of gastritis cystica profunda. J Gastrointestinal Surg.

[9] Roepke TK, Purtell K, King EC, La Perle KMD, Lerner DJ, Abbott GW (2010). Targeted deletion of Kcne2 causes gastritis cystica profunda and gastric neoplasia. PLoS One.

[10] Deng S, Cao Y, Shen L (2019). Bile reflux gastritis cystica profunda: A case report and literature review. Medicine (Baltimore).

[11] Okada M, Iizuka Y, Oh K, Murayama H, Maekawa T (1994). Gastritis cystica profunda presenting as giant gastric mucosal folds: the role of endoscopic ultrasonography and mucosectomy in the diagnostic work-up. Gastrointest Endosc.

[12] Park JS, Myung SJ, Jung HY (2001). Endoscopic treatment of gastritis cystica polyposa found in an unoperated stomach. Gastrointest Endosc.

[13] Seevaratnam R, Cardoso R, McGregor C (2012). How useful is preoperative imaging for tumor, node, metastasis (TNM) staging of gastric cancer? A meta-analysis. Gastric Cancer.

[14] Matsumoto T, Wada M, Imai Y, Inokuma T (2012). A rare cause of gastric outlet obstruction: gastritis cystica profunda accompanied by adenocarcinoma. Endoscopy.

[15] Butt MO, Luck NH, Hassan SM, Abbas Z, Mubarak M (2015). Gastritis profunda cystica presenting as gastric outlet obstruction and mimicking cancer: a case report. J Transl Int Med.

[16] Park WY, Lee SJ, Kim YK (2018). Occurrence of metachronous or synchronous lesions after endoscopic treatment of gastric epithelia dysplasia—impact of histologic features of background mucosa. Pathol Res Pract.

[17] Qizilbash AH (1975). Gastritis cystica and carcinoma arising in old gastrojejunostomy stoma. Can Med Assoc J.

[18] Bechade D, Desrame J, Algayres JP (2007). Gastritis cystica profunda in a patient with no history of gastric surgery. Endoscopy.

[19] Dikicier E, Altintoprak F (2018). Multiple gastritis cystica profunda in a patient without gastric surgery history. Arch Iran Med.

[20] Yu XF, Guo LW, Chen ST, Teng LS (2015). Gastritis cystica profunda in a previously unoperated stomach: a case report. World J Gastroenterol.

[21] Allen JR, Norback DH (1973). Polychlorinated biphenyl- and triphenyl-induced gastric mucosal hyperplasia in primates. Science.

[22] Mitomi H, Iwabuchi K, Amemiya A, Kaneda G, Adachi K, Asao T (1998). Immunohistochemical analysis of a case of gastritis cystica profunda associated with carcinoma development. Scand J Gastroenterol.

[23] Iwanaga T, Koyama H, Takahashi Y, Taniguchi H, Wada A (1975). Diffuse submucosal cysts and carcinoma of the stomach. Cancer.

[24] Lee MS, Cho BS, Park JS, Koo HC, Han HY, Kang DW (2013). Premalignant lesion of heterotopic pancreas combined with gastritis cystica profunda in gastric fundus. J Gastrointestin Liver Dis.

[25] Carvalho JR, Quadros CA, Meireles L (2018). Gastritis cystica profunda mimicking a GIST—a diagnostic challenge. Gastroenterol Hepatol.

[26] Wang L, Yan H, Cao DC et al (2014) Gastritis cystica profunda recurrence after surgical resection: 2-year follow-up. World J Surg Oncol 12:133 10.1186/1477-7819-12-133 PMC4030027 24885818

[27] Moon SY, Kim KO, Park SH (2010). Gastritis cystica profunda accompanied by multiple early gastric cancers. Korean J Gastroenterol.

[28] Tomizuka T, Mazaki T, Mado K (2008). A case of gastritis cystica profunda. Surgery.

[29] Yang DM, Jung DH, Kim H (2004). Retroperitoneal cystic masses: CT, clinical, and pathologic findings and literature review. Radiographics.

[30] Machicado JD, Thosani N, Quesada A (2012). Gastritis cystica profunda: a rare tumor mimicking malignancy. Am J Gastroenterol.

[31] Tan Y, Hu C, Liu D (2016). An unusual gastric mass gastritis cystica profunda. Gastroenterology.

[32] Kurland J, DuBois S, Behling C, Savides T (2006). Severe upper-GI bleed caused by gastritis cystica profunda. Gastrointest Endosc.

[33] Tang L, Li ZY, Li ZW (2015). Evaluating the response of gastric carcinomas to neoadjuvant chemotherapy using iodine concentration on spectral CT: a comparison with pathological regression. Clin Radiol.

[34] Hur S, Han JK, Kim MA, Bae JM, Choi BI (2010) Brunner’s gland hamartoma: computed tomographic findings with histopathologic correlation in 9 cases. J Comput Assist Tomogr 34:543–547 10.1097/RCT.0b013e3181d472dc 20657222

[35] Mehren M, Joensuu H (2018). Gastrointestinal stromal tumors. J Clin Oncol.

[36] Gossios K, Katsimbri P, Tsianos E (2000). CT features of gastric lymphoma. Eur Radiol.

[37] Hu YB, Gui HW (2019). Diagnosis of gastric duplication cyst by positron emission tomography/computed tomography: a case report. World J Clin Cases.

